# Implementation of QbD strategies in the inoculum expansion of a mAb production process

**DOI:** 10.1002/elsc.202000056

**Published:** 2020-12-03

**Authors:** Ole Jacob Böhl, Jana Schellenberg, Janina Bahnemann, Bernd Hitzmann, Thomas Scheper, Dörte Solle

**Affiliations:** ^1^ Leibniz Universität Hannover Institut für Technische Chemie Hannover Germany; ^2^ Universität Hohenheim Fachgebiet für Prozessanalytik und Getreidewissenschaft Stuttgart Germany

**Keywords:** CHO, inoculum expansion, mAb, PAT, QbD

## Abstract

The quality by design approach was introduced to the biopharmaceutical industry over 15 years ago. This principle is widely implemented in the characterization of monoclonal antibody production processes. Anyway, the early process phase, namely the inoculum expansion, was not yet investigated and characterized for most processes. In order to increase the understanding of early process parameter interactions and their influence on the later production process, a risk assessment followed by a design of experiments approach was conducted. The DoE included the critical parameters methotrexate (MTX) concentration, initial passage viable cell density and passage duration. Multivariate data analysis led to mathematical regression models and the establishment of a designated design space for the studied parameters. It was found that the passage duration as well as the initial viable cell density for each passage during the inoculum expansion have severe effects on the growth rate and viability of the early process phase. Furthermore, the variations during the inoculum expansion directly influenced the production process responses. This carry‐over of factor effects highlights the crucial impact of early process failures and the importance of process analysis and control during the first part of mAb production processes.

**Abbreviations**
CHO: Chinese hamster ovary cells
FMA: feed medium A
FMB: feed medium B
FMEA: failure mode and effect analysis
mAb: monoclonal Antibody
MLR: multiple linear regression
MTX: methotrexate
MVDA: multivariate data analysis
PM: production medium
QbD: quality by design
RPN: risk priority number
SM: stock medium
VCD: viable cell density

## 1 INTRODUCTION

The Quality by Design (QbD) principle is a risk‐based approach to pharmaceutical development and manufacturing [1]. It emerged in 2004 with the *Current Good Manufacturing Practice for the 21^st^ century* initiative [2]. Based on this, the United States Food and Drug Administration (FDA) launched a guidance protocol for the biopharmaceutical industry. This guidance consists of the report *PAT – A framework for Innovative Pharmaceutical Manufacturing and Quality Assurance* [3] and the International Conference of Harmonisation (ICH) guidelines *Q8 Pharmaceutical Development*, *Q9 Quality Risk Management* and *Q10 Pharmaceutical Quality Systems* [4, 5, 6].

**PRACTICAL APPLICATION**

The inoculum expansion was assessed as a critical part of the mAb production process by examination of critical process parameters in a design of experiments approach. Our research resulted in the definition of a designated design space and new feedback strategies for a robust process control. This highlights the importance of early process understanding and investigation, while marking the first step towards a full process characterization regarding quality by design principles. Implementing the conducted concept in the process development was thereby shown to be crucial for the studied process as well as similar cell culture processes.

**FIGURE 1:**
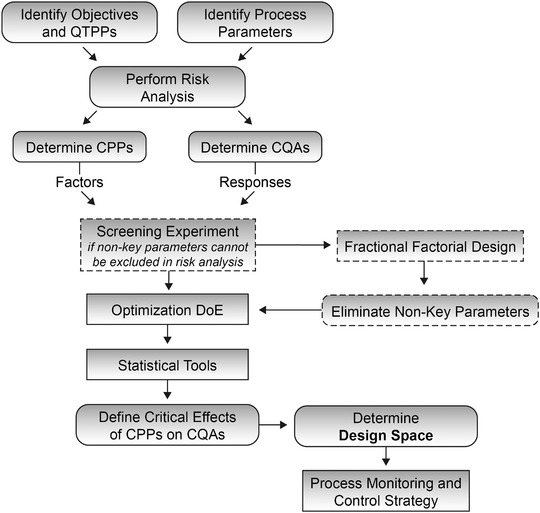
Schematic strategy for implementation of QbD and determination of a process design space in a biopharmaceutical production process. CPP, critical process parameter; CQA, critical quality attribute; DoE, design of experiments; QTPP, quality target product profile

Before introducing this guidance the pharmaceutical industry used a traditional approach for process development and regulation: A rigid manufacturing process with batch to batch quality controls and no methodical connection between process, product and clinical application. The QbD paradigm, imposed by the ICH guidelines, was defined as “a systematic approach to development that begins in predefined objectives and emphasises product and process understanding and process control based on sound science and quality risk management” [4]. The objective of this approach is to “build quality into the product” rather than “testing quality into the product” [7, 8]. Thereby manufacturers can reduce the post‐commercial filings for process changes, leading to greater flexibility in biopharmaceutical production and quality control. A general roadmap for successful implementation of the QbD philosophy is shown in Figure 1.

The streamlined operation starts with the product and process definition, identification of process parameters and a general risk assessment, often using the Failure Mode and Effect Analysis (FMEA) [9, 10]. Subsequent process characterizations are typically performed in qualified scaled‐down models, using a Design of Experiments approach to set up conclusive experiments [11, 12]. An analysis of the compiled results leads to the definition of a designated Design Space [13]. The FDA defines Design Space as “the multidimensional combination and interaction of input variables that have demonstrated to provide assurance of quality” [4].

Thereby, design space can be described as the acceptable range of process parameters, which is located in a broad characterisation range and inherits the operating range with optimal set points. Working within the defined design space is therefore not considered to be a process change and hence needs no further validation, resulting in cost savings and the maintenance of a steady workflow.

**FIGURE 2:**
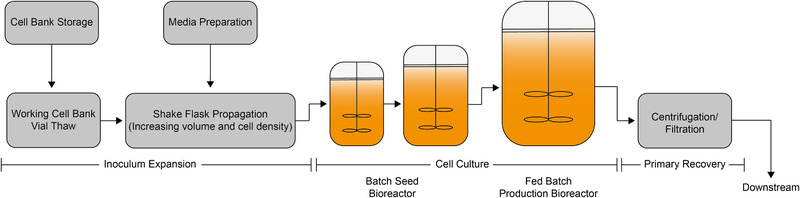
Schematic representation of the process workflow of monoclonal antibody cell cultivation in larger scale

This work will focus on the QbD implementation of a monoclonal Antibody (mAb) production process using a Chinese Hamster Ovary (CHO) cell cultivation process [14, 15]. Concrete QbD case studies, like the CMC Biotech Working Group for mAb production processes and the following downstream part are published [16]. Yet, reports on the QbD implementation on early process steps, namely the inoculum expansion, are still limited. It consists of multiple cell passages, which involve the transfer of a defined cell number into fresh culture medium. By passaging with specific intervals the inoculums is expanded, while maintaining the cells in a healthy and actively growing state [17]. Thereby the predefined cell concentration for inoculation of the production process is achieved. This early phase of said processes is still not as frequently monitored and regulated as the production part in larger bioreactors and often falsely treated as a “black box” with no crucial impact on the following process. The critical impact of early phase parameters was not yet investigated thoroughly and is therefore not fully understood. As envisioned by the ICH guidelines, the QbD philosophy should be applied throughout the entire lifecycle of a biopharmaceutical product [4, 18]. This work will therefore create a linkage between process parameters of the inoculum expansion and process performance of the production process. A schematic overview of the process is shown in Figure 2.

## 2 MATERIALS AND METHODS

### 2.1 Cell line and material

For this study a DG44 CHO suspension cell line producing an IgG1 monoclonal Antibody (mAb) was used (Sartorius Stedim Cellca GmbH). Cells were cultivated in shake flasks (Corning, USA) in a Heracell 240 CO_2_ Incubator (Thermo Scientific, USA) using a MaxQ CO_2_ Plus shaker platform (Thermo Scientific, USA). The cultivation was performed in proprietary and chemically defined cell culture media, all part of the Sartorius Stedim Cellca medium platform. The inoculum expansion was performed using stock medium (SM), whereas the production process used production medium (PM). Additionally, two feeds were used during the production process. Macro nutrients (e.g. glucose) were added using feed medium A (FMA), while micro nutrients (e.g. amino acids) were supplemented using feed medium B (FMB) (Sartorius Stedim Cellca GmbH, Germany).

For the first passage of inoculum expansion a cryo vial, containing 1 mL CHO suspension (30 × 10^6^ cells/mL), was thawed and transferred to a 15 mL Falcon tube (Sarstedt, Germany) with 10 mL stock medium. The suspension was centrifuged at 190 x g for 3 min using a Microstar17 centrifuge (VWR International, USA). After removal of the supernatant, the pellet was resuspended in 10 mL pre‐warmed (36.8°C) stock medium and transferred to a 500 mL shake flask with 140 mL pre‐warmed stock medium. The inoculum expansion was conducted over 16 days at 36.8°C and 7.5% CO_2_ with a shaking rate of 120 rpm. Cells were passaged by centrifugation of the needed amount of cell suspension, removal of the supernatant and resuspension in fresh stock medium to the initial passage viable cell density (VCD). During the first three passages, the required amount of methotrexate (MTX) was added to the stock medium. From the second passage, 125 mL shake flasks with a working volume of 25 mL were used for the experiments.

The production process was performed in 125 mL shake flasks with 25 mL pre‐warmed production medium. All production cultivations were inoculated to an initial viable cell density of 0.3 × 10^6 ^cells/mL and run over 8 days with daily feeds (1% feed medium A, 0.1% feed medium B) from day 3. Due to the cultivation in shake flasks the pH and the dissolved oxygen were not controlled, yet the process was previously analyzed and shown to be reproducible and comparable to bioreactor cultivations of different scales. To improve the comparability all experiments were simultaneously conducted in the same incubator with identical process conditions.

### 2.2 Analytics

Samples were taken daily during the inoculum expansion as well as during the production process. Viable cell densities and viabilities were measured using a Cedex HiRes (Roche Innovatis, Swiss) with a 1:2 dilution in 10 % phosphate‐buffered saline (PBS). Growth rates of the production process were calculated by linear regression of the natural logarithm of viable cell densities against culture duration. The inoculum growth rate relates to the respective last passage of every inoculum expansion (N‐1‐step), in order to ensure optimal comparability of the examined factor effects. Viabilities were calculated as average over the culture duration for the inoculum expansion as well as the production process. The mAb titer was analyzed during the production process by using the Cedex Bio (Roche, Swiss). Therefore, cell separation was performed by centrifugation for 5 min at 190 x g. Specific antibody titers were calculated by dividing the final antibody titer by the integral viable cell concentration over the entire production process duration.

### 2.3 Failure mode and effect analysis (FMEA)

The risk assessment was performed using the FMEA approach [9]. The FMEA divides the potential risk into three main factors. The Probability (P) evaluates the likelihood of a possible failure mode. Failure impact on the system and the product are assessed by the Severity (S). In addition, the Detection (D) assesses the possibility to detect and correct a failure mode. These factors were rated on a scale between 1 and 5 individually for each process parameter. Multiplication of these ratings resulted in the Risk Priority Number (RPN) for each respective parameter.

### 2.4 Design of experiments (DoE)

The design and analysis was performed using the DoE software MODDE 12 (Umetrics, Sartorius Stedim Data Analystics, Germany). The three parameters with the highest RPN during the risk assessment were used as factors (F_1 _= MTX concentration, F_2 _= passage duration, F_3 _= initial passage viable cell density) for a central composite design with three centre‐point runs. The resulting design is illustrated in the supplements. Hereinafter, the factor settings are described as 0 for centre‐point level and –1/1 for the low and high levels of the full factorial cube, respectively. The additional levels for the star of the central composite design are described as –2 and 2.

The initial viable cell density of each passage was varied equally between 0.1 and 0.3 × 10^6^ cells/mL with a step size of 0.05 × 10^6^ cells/mL, while the MTX concentration was varied between 0 and 30 nM with a step size of 7.5 nM. The passage duration was varied without any additional level between 2, 3/4 and 5 days with a constant total inoculum expansion time of 16 days. A passage duration lower than –1 level was not possible, because shorter passages than 2 days did not result in the minimum viable cell number needed for sufficient inoculum expansion. Also the total duration of all experiments was preserved at 16 days. According to the process development, this is the time needed to expand the inoculum to the minimum viable cell number for inoculation of larger production bioreactors. Therefore, a plane star was applied in this design, which resulted in a total of 15 runs.

All 15 runs were cultivated simultaneously in the same incubator. To improve the comparability of the runs, one vial thaw and centre‐point passage one was used to inoculate the following 15 experiments under described levels. The factor changes were implemented on every seed step from the second passage onwards. Also, all runs respective last passage was run over 3 days, to compare the final growth rate and enable production process inoculation on the same day. Passage duration centre‐point runs were passaged every 3‐4 days, resulting in a total of five inoculum expansion steps. The –1 level runs were passaged every 2 days, the 1 level runs every 5 days, resulting in a total of 7 and 4 passages respectively (see Figure 4). An overview of the experimental set up and the explained numerical coding of the parameter levels are depicted in the supplements. Six different responses were analysed: Inoculum viability, inoculums growth rate, production viability, production growth rate, total mAb titer and specific mAb titer. The responses were predicted by using the following model:

$$\begin{array}{ccc} \;R_{i} & = & p_{0}\;+p_{1}F_{1}+p_{2}F_{2}+p_{3}F_{3}+p_{4}F_{1}^{2}+p_{5}F_{2}^{2}+p_{6}F_{3}^{2}\\ & + & p_{7}F_{1}F_{2}+p_{8}F_{1}F_{3}+p_{9}F_{2}F_{3} \end{array}$$

Here, $R_{i}$ is one of the six responses, p_0_ is the intercept, p_j_ are the coefficients of the factors F_1_, F_2_ and F_3_. Factors whose coefficient has the value zero in its confidential interval are regarded to have no significant influence on the response and are therefore removed from the model.

### 2.5 Multivariate data analysis (MVDA)

Multiple linear regression (MLR) was used to fit the mathematical model. Various model statistics were investigated in order to evaluate the conducted model. The R‐squared (*R* ^2^) term is the fraction of the variation of the response explained by the model. The adjusted R‐squared (*R* ^2^_adj_) term is adjusted for the degrees of freedom of the model.

$$\;R^{2}=\;1-\frac{\sum_{i\;=\;1}^{n}{\left(y_{i}-{\hat{y}}_{i}\right)}^{2}}{\sum_{i\;=\;1}^{n}{\left(y_{i}-\bar{y}\right)}^{2}}$$

$$\;R_{adj}^{2}=\;1-\left(1-R^{2}\right)\frac{n-1}{n-p}$$

Here, $y_{i},\;{\hat{y}}_{i},\;\mathrm{and}\;\bar{y}$ are the measured data, the prediction of calibration data and the mean of the measured data, respectively; n is the number of measurement points and p the number of parameters. Statistical accuracy of future predictions of the model is estimated by the Q‐squared (*Q* ^2^) term. If the y data are not used for calibration, then the *Q*² is calculated as follows.

$$\;Q^{2}=\;1-\frac{\sum_{i\;=\;1}^{n}{\left(y_{i}-{\hat{y}}_{i}\right)}^{2}}{\sum_{i\;=\;1}^{n}{\left(y_{i}-\bar{y}\right)}^{2}}$$

The model p‐value represents the significance of the fitted model. It tests the null hypothesis, that the coefficient is equal to zero, for each term. Here, the p‐value belongs to an f‐test, which compares an intercept‐only model (just a constant y = const) with the model under consideration ($R_{i}$). The tested null hypothesis is that a fit of the intercept‐only model and the model $R_{i}\;$are equal. The reproducibility r compares the replicate variability to the overall variability of the experimental data.

$$r\;=\;1-MS_{pe}/MS_{tot}$$

Here, $MS_{pe}$ is the mean square of the pure error and $MS_{tot}$ the total mean square of y. A reproducibility value of 1 signifies perfect reproducibility.

## 3 RESULTS AND DISCUSSION

In order to investigate possible influences of the inoculum expansion on the production process, critical process parameters must be identified and assessed based on a design of experiment to establish a design space for this early process step. During this evaluation the effects of the inoculum expansion on the production process must be considered as target values for a high quality process.

### 3.1 Risk assessment

**FIGURE 3:**
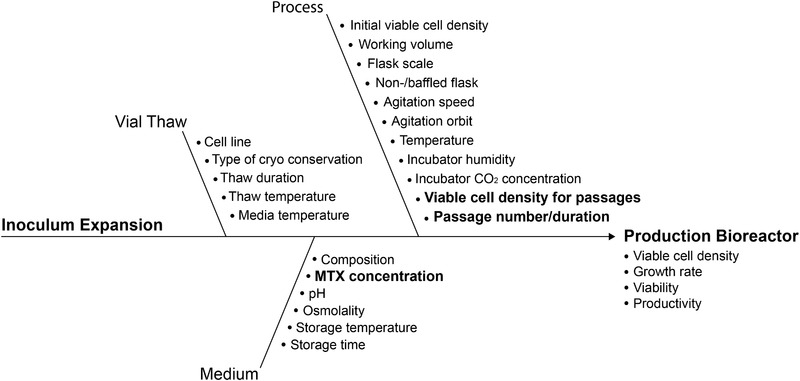
Ishikawa diagram for the inoculum expansion. Bold parameters were determined as critical and therefore examined during this study

All possible process parameters were listed in an Ishikawa diagram (Figure 3), which depicts potential root causes for specific failure events during the studied process. The parameters were categorized in different parts of the inoculum expansion, namely the vial thaw, the medium and the process.

Historical data as well as profound process understanding were used to rank process parameters according to their potential risks for the process robustness and productivity. Hence, a Failure Mode and Effect Analysis approach was used. The probability, severity and detectability of each parameter were rated individually and multiplied, resulting in the respective risk priority number.

Some specific parameters, like general media composition and cell line, were not taken into account during the risk assessment because they were profoundly analysed and optimized during process and media development. Others like the temperature and incubator CO_2_ concentration were ruled out during risk assessment due to the optimized control of the used incubator. This results in low failure probability and high detectability of possible failure modes like loss of CO_2_ pressure. Since the process was shown to be replicable in scaled down bioreactors, parameters like the flask scale were also known to be non‐critical for the process [19]. The threshold RPN to consider a parameter to be critical for the system‐ and product robustness was determined to be 15, representing a moderate to significant risk of at least two of the FMEA factors. Critical process parameters identified in this way are shown in bold in Figure 3 and are represented in Table 1. The complete FMEA table is summarized in the supplements.

**TABLE 1:** Failure mode and effect analysis (FMEA) results for the examined parameters

	Probability	Severity	Detection	RPN
MTX concentration	1	5	4	20
Passage duration	3	3	2	18
Initial passage VCD	2	2	4	16

Probability, severity and detection were individually rated between 1 and 5. In words, 1 represents no potential risk, 3 moderate/controllable risk and 5 significant risk

The methotrexate concentration has a significant influence on the antibody production and severe toxic effects on the CHO cells (Severity = 5) [20]. A failure in adjusting the MTX concentration within the stock medium is furthermore not easily detectable during the later process steps (Detection = 4). Therefore, the MTX concentration was considered a critical parameter and used as factor F_1_ in the DoE, despite the low failure probability (Probability = 1) due to standard operation procedure and good laboratory manufacturing practise.

For the passage duration (F_2_), the balance between too short passages, leading to an insufficient viable cell density for the expansion of the seed steps, and too long passages, leading to consumed nutrients, can be a critical aspect (Severity = 3). Also this parameter can be of special interest because other failures in the system, e.g. the preparation of the production bioreactor, can lead to a delay in the process and longer passage duration. Likewise, the tight schedule in production and a failure in seed culture could lead to shortened time available for the new inoculum expansion (Probability = 3). In reality, the layout of the inoculum expansion can also be dependent on other factors like working hours and days in the industry. Therefore, the understanding of the impact of the passage duration in combination with the other critical process parameters is needed to define a robust design space for the inoculum expansion.

Failures in the initial passage viable cell density (initial passage VCD) would be detected quickly; however, a readjustment is virtually impossible without time consuming preparation of new shake flasks and medium (Detection = 4). The readjustment of the passage viable cell density is also logistically impossible in a scaled‐up approach with seed bioreactors due to the preparation of the needed reactor. The probability of a failure was considered to be low yet possible, due to rare inaccuracies during the passages (Probability = 2). It was also considered to show a direct non‐severe impact on the nutrient consumption and thereby the growth rate and viability (Severity = 2). Therefore, the initial passage VCD is used as factor F_3_.

### 3.2 DoE concept and implementation

The ICH Guidelines determined the implementation of a Design of Experiments approach to investigate the single and combined influence of critical process parameters. Accordingly, the MTX concentration (F_1_), the passage duration (F_2_) and the initial passage viable cell density (F_3_) were combined in a central composite DoE.

**FIGURE 4:**
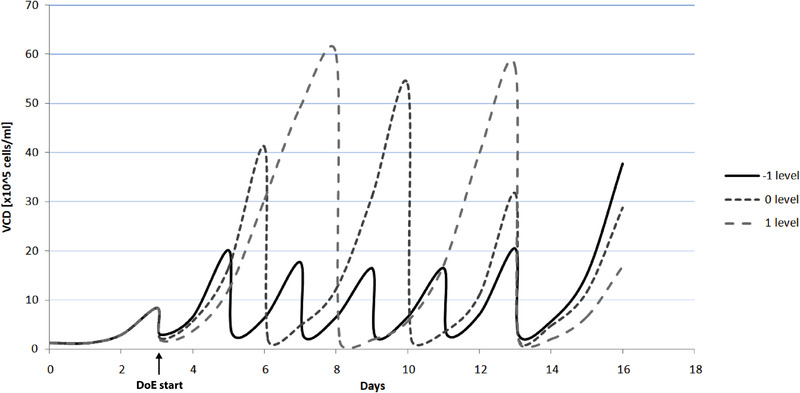
Viable cell densities during the inoculum expansion for experiments with –1/0/1 levels for the passage duration. Cells were split to 0.2 × 10^6^ cells/mL for each passage

The initial passage VCD as well as the MTX concentration were varied on a five level scale (–2,–1,0,1,2), while the passage duration could only be varied on three levels (–1,0,1). As explained in the Material and Methods, a passage duration lower than 2 days (–1 level) did not result in the minimum viable cell number needed for sufficient inoculum expansion. Longer passages than 5 days (1 level) did not fit in the experimental design and total inoculum expansion duration of 16 days. Furthermore, previous experiments show that inoculum expansion passages of more than 5 days result in severe negative effects due to consumed nutrients. The centre‐point experiment on level 0 was conducted with four passages of 3 days and one passage of 4 days to fulfil the total duration of 16 days. The respective last passage of every experiment was cultivated 3 days before inoculation of the production processes. Figure 4 visualizes the passage duration principle by plotting the viable cell densities of three different runs (–1, 0, 1).

During the inoculum expansion the growth rates and cell viabilities of each experiment were monitored as responses for the early process steps. The growth rate was calculated for the respective last passage of the inoculum expansion. These responses provide specific information on the inoculum expansion culture. The growth rate determines when the minimum viable cell number for inoculation of the next passage or the production bioreactor will be reached. An inoculum expansion with a high proliferation rate also shortened the lag phase of the production process. Therefore, improvements in the inoculum growth rate could lead to reduced process time and production costs. A maximized viability during the inoculum expansion is crucial for the maintenance of the culture and the successful inoculation of the production bioreactor.

In order to investigate the influence of the determined inoculum expansion process parameters on the later process steps, all inoculum cultures were used to inoculate scaled‐down production processes. These were simultaneously cultivated at standard conditions and sampled daily. The growth rates, average viabilities and the antibody titer were monitored as responses for the later state of the process. To obtain an optimal production process all of these responses should be maximized. A high growth rate leads to an early reach of an acceptable VCD for sufficient antibody production rates. An optimal viability ensures the maintenance of the culture during the entire production process. Moreover, the high viability is important to minimize the amount of cell debris, DNA and HCPs that are released to the medium and have to be removed expensively during the downstream part. To provide a profitable process the final product titer obviously has to be maximized. Additionally to the antibody titer, the specific titer was investigated as a response. It elucidates the cell specific productivity for the studied culture conditions. Thereby, it is possible to establish a specific production control between optimized growth and productivity. Typically, quality attributes in the biopharmaceutical industry include the quality of the product, such as the bioactivity or the glycosylation of the antibody. Yet, we concluded that the quality attributes for the inoculum expansion in our experimental set up have to be the condition of the final inoculum. That includes the growth rate, viability and also the productivity of the following production process. These were rated as sufficient responses for a meaningful conclusion regarding the inoculum expansion performance and its direct influence on the production.

### 3.3 Multivariate data analysis

**FIGURE 5:**
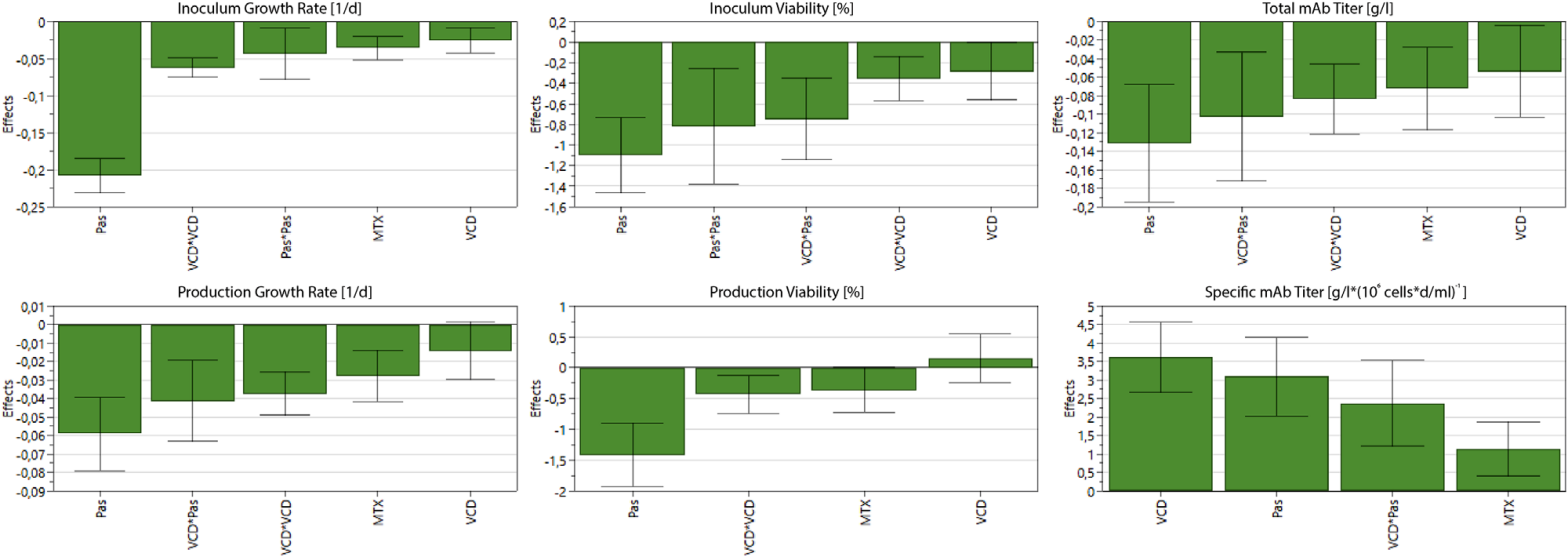
Factor (parameter) and its interaction effect plots for the inoculum expansion and production process responses. Not shown parameters as well as parameters whose confidential interval includes zero were determined to have no significant effect on the respective response (Pas, passage duration, MTX, TX concentration, VCD, initial passage viable cell density)

Multiple linear regression (MLR) was used to analyse the factors and evaluate the responses (inoculums viability, inoculums growth rate, production viability, production growth rate, total mAb titer and specific mAb titer) by fitting mathematical models. The regression models were used to determine effects of the studied factors (process parameters) and their interactions. Factors, which include zero in its confidential interval, are insignificant and hence eliminated from the corresponding model. The resulting main effects are plotted in Figure 5. Most of the parameters and parameter interactions show negative coefficients for the prediction of the growth rates and viabilities of the inoculum expansion and the production process as well as the total mAb titer. The passage duration shows the most considerable influence, both as a linear effect (Pas) for all responses as well as a non‐linear effect (Pas*Pas) for the inoculum responses. Furthermore, it shows strong effects in the interaction with the initial passage viable cell density (VCD*Pas). Its negative coefficient on the growth rate and viability can be explained by the consumption of important nutrients as well as the aggregation and stress of cells at higher cell densities during the inoculum expansion. The fact that those effects are still detectable in the production process, supports the hypothesis that parameter effects from the early process steps indeed carry over to the production process. This certainly shows the severe influence of the inoculum expansion on later process steps. A slightly lower but still significant negative coefficient on all responses was observed for the initial passage viable cell density (VCD), which can also be explained by its strong connection to the nutrient consumption and cell interaction from the beginning of every passage. The MTX concentration (MTX) only showed negative linear effects on the production process responses, which again underlines the importance of early process monitoring and control strategies.

Negative coefficients regarding the total mAb titer can be explained by the connection between viable cell density and total product concentration. Evidently, a higher cell count during the cultivation leads to an increased titer. Therefore, the explained negative effects on the growth rate influence the total mAb titer, which is why the specific mAb titer was calculated and used as an additional response. In contrast to the discussed responses, the studied parameters show consistently positive effects on the cell specific mAb titer. This could signify that cells, growing under suboptimal cultivation conditions in regard to growth and viability, show an increased specific antibody production. Again, the passage duration (Pas) and its interaction with the initial passage viable cell density (VCD*Pas) show the most significant effects, supporting the concept of counteraction between optimal growth and specific productivity. Also, the MTX concentration (MTX) shows a comparatively high effect on the specific mAb titer, indicating the importance of balance between selection pressure and ideal cultivation conditions.

**FIGURE 6:**
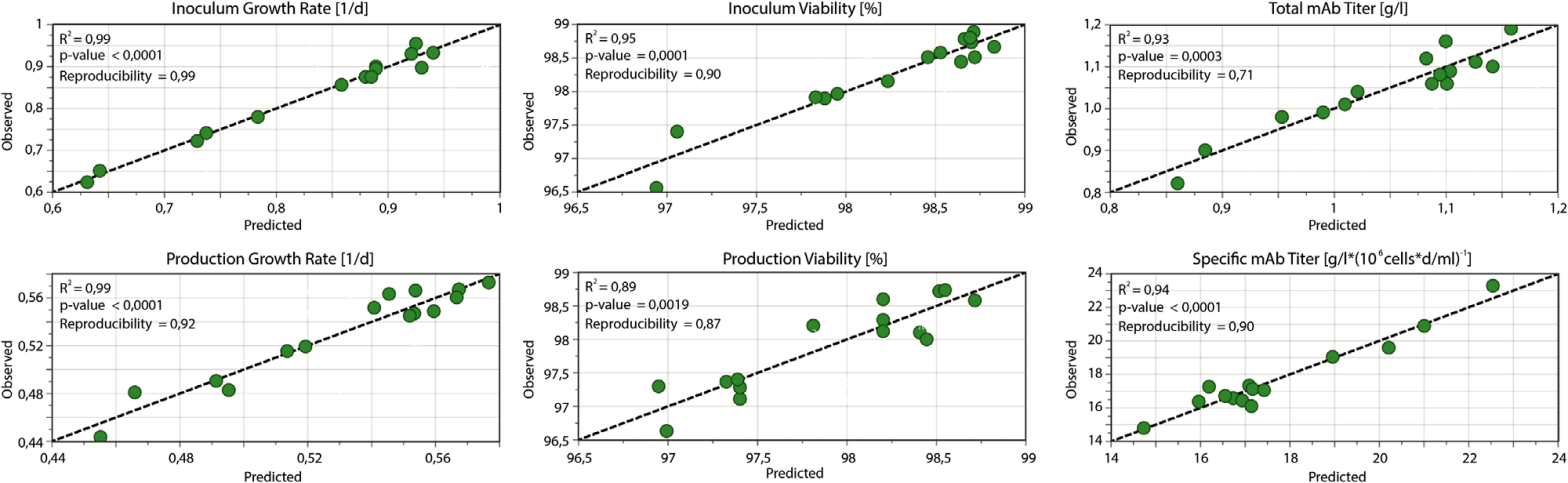
Observed versus predicted plots for the inoculum expansion and production process responses

To investigate the meaningfulness of the regression models, various model statistics were analysed. The full model statistics table is shown in the supplements. All models show a good fit to the experimental data with *R^2^* and *R^2^_adj_* values mostly over 0.9. The *Q^2^* term, representing the predictive power of the model and its ability to generalize to unseen data, is also sufficient for all responses with the lowest value for the production viability at 0.62. Model significance, represented by the p‐value, shows a great meaningfulness for all responses with values close to zero. Also the reproducibility of the model is ensured for all responses with reproducibility values all over 0.7. In summary, the model statistics elucidate well fitted regression models for all selected responses. Also, the predictive power as well as the reproducibility is sufficient for the analysis of parameter effects. Since the model shall be used for future predictions and the establishment of a design space, the predictive power is particularly important. Thus, the observed response values were plotted against the predicted response values, to enable further insights on the model and possible outliers. The resulting observed vs predicted plots for all responses are shown in Figure 6.

For the evaluated responses the plots show an overall strong predictive power. Furthermore, this plot allows an explanation for the comparatively low model statistic values of the production viability. It can show possible individual outliers as well as the biggest overall spread of data points. These were further investigated by analysis of the response residuals and the normal probability of distribution, which implied no significant outliers. In summary, the models were verified by analysis of the model statistics and the observed vs. predicted plot. Overall well fitted models with strong predictive power and a great reproducibility were determined.

**FIGURE 7:**
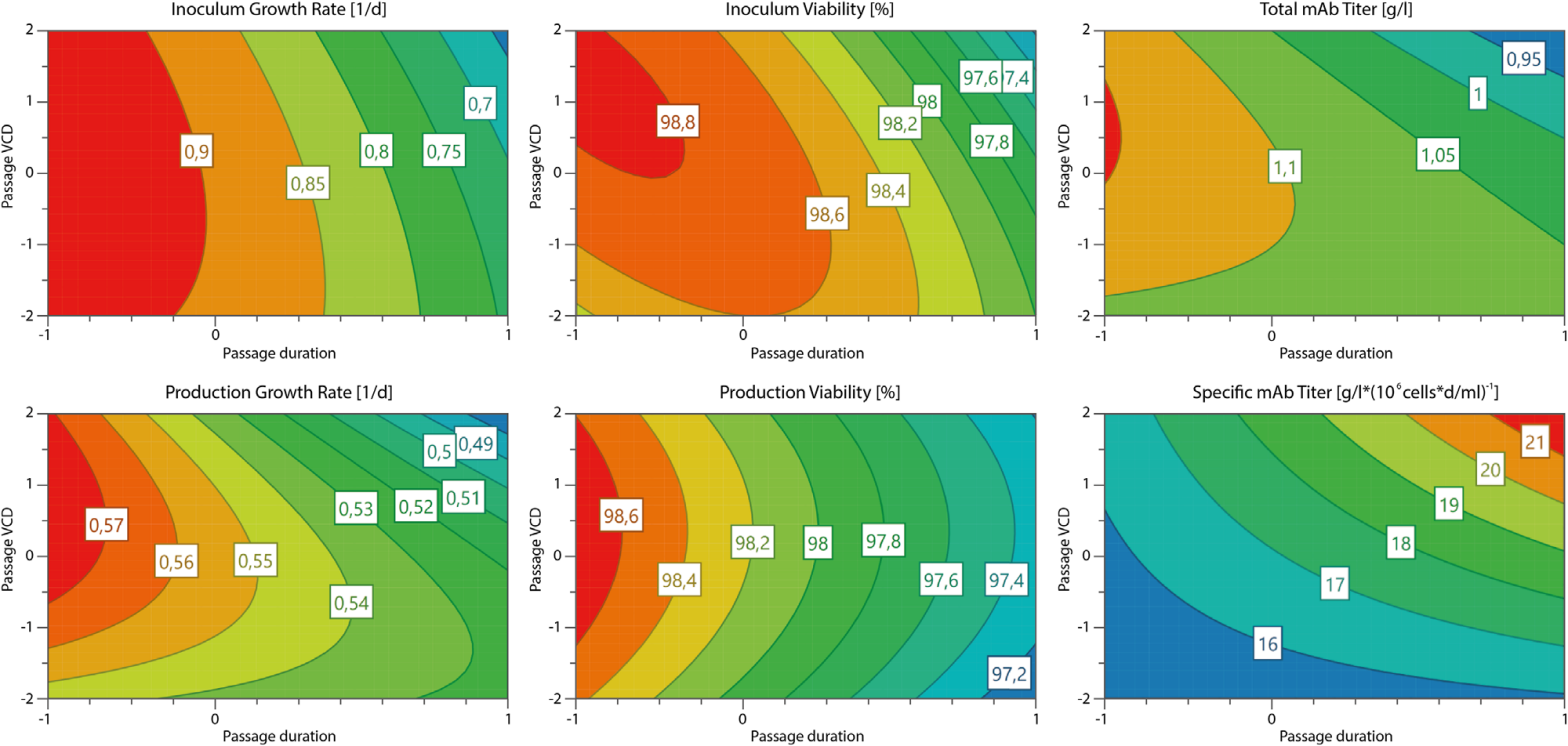
Response contour plots showing results for inoculum expansion and production process responses

The observed interaction effects of the passage duration and the initial passage VCD were further investigated by analysis of the response contour plots shown in Figure 7. These provide a two‐dimensional illustration of the combined parameters and their impact on the respective response. All represented growth rates and viabilities as well as the total mAb titer show similar contour plots. The estimated optimum for these responses is close to the –1 level for the passage duration and slightly higher than the 0 level for the initial passage VCD. Passage durations lower than –1 level were practically not possible because passages shorter than 2 days did not result in the minimum viable cell number needed for sufficient inoculum expansion. That is why the passage duration levels could not be shifted further towards the estimated optimum. A passage duration longer than the 0 level resulted in worsened response values for all said responses.

Furthermore, the non‐linearity as well as the interaction of the parameter effects, which were observed in the effect plot, is detailed in the contour plots. This can be seen particularly in the inoculum viability contour plot with an anticipated optimum at –1 level for the passage duration and 1 level for the passage VCD. The presence of a predictable optimum underlines the effect interaction of factors. Shown similarities between the inoculum expansion and the production process responses also underline the hypothesis that the early process steps have a considerable influence on the production process. These investigations highlight the importance of a sound monitoring and control strategy for the shown parameters.

The total mAb titer was shown to be the most robust response in relation to the passage duration and the initial passage VCD. Again, the optimum was anticipated below –1 level for the passage duration. Besides that, the contour plot shows a substantial robust working area with no considerable loss in antibody titer. That is likely due to the cell line and process development, which was focused on the optimization of the produced antibody titer.

In contrast, the specific antibody titer shows an estimated optimum at the upper end of the examined space. Increasing passage duration as well as increasing initial passage viable cell density equally improved the specific productivity of the cells. Anyway, the increased productivity could still not compensate the negative effects on the growth rate and therefore the total cell number and final product titer. Taking only the studied factor effects into account, it was shown to be yet more worthwhile to improve the growth and viability of the cells in order to increase the total mAb titer.

**FIGURE 8:**
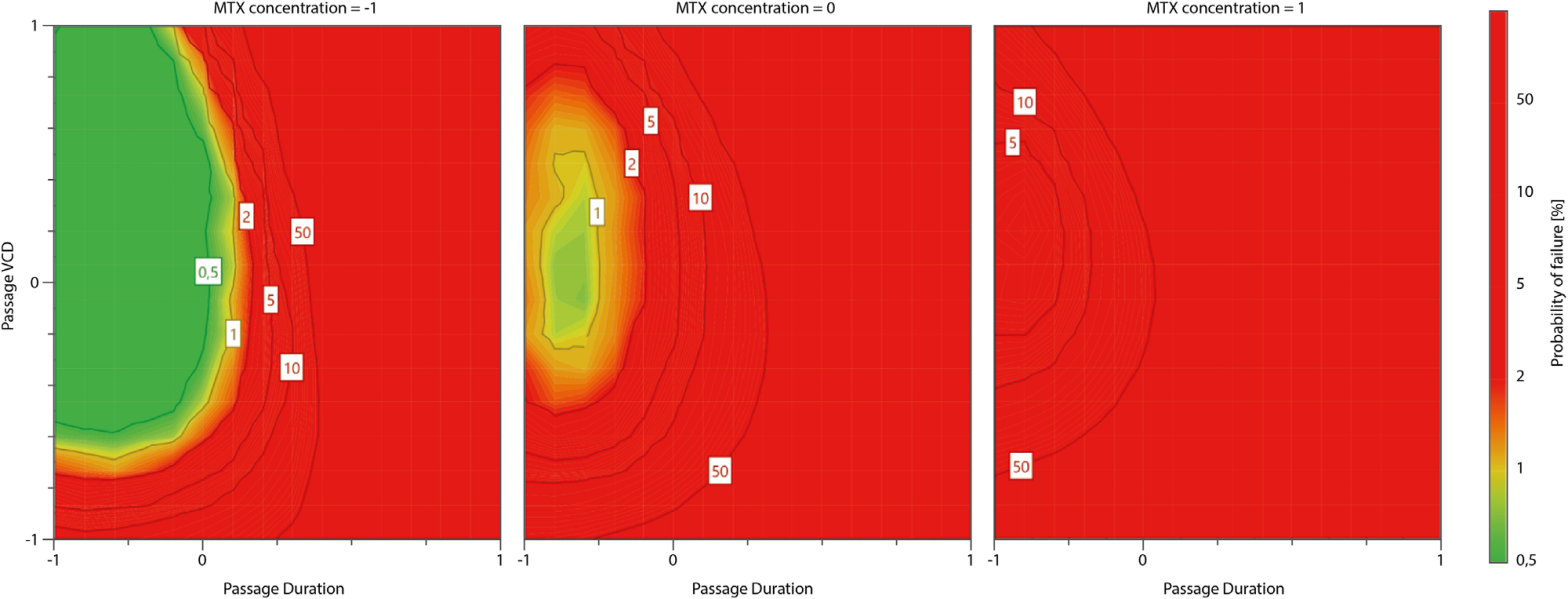
Determined design space for the inoculum expansion and production process viability/growth rate and the antibody titer with colour coded probability of failure

Therefore, the respective growth rate and viability responses as well as the total mAb titer response were used for further investigation. On that basis a design space for the studied process was calculated. Monte Carlo simulations were used to compile the needed probability statistics. The resulting design space is shown in Figure 8. It visualizes the region in the experimental design where all response specifications are fulfilled with given probabilities. These specifications were experimentally accounted and adjusted with profound process knowledge which was expanded during this study.

A robust design space, marked in green, was determined around the –1 level for the passage duration and 0 level for the initial passage viable cell density. Increasing MTX concentration shows a negative effect on said design space. The set standard operation for the passage duration is located at the boundary of the acceptable probability values. Consequently, it would be beneficial for the process safety to either decrease the MTX concentration to broaden the design space or to shorten the passage duration to shift the process further towards the design space midpoint. Mathematical optimization of the three parameters resulted in the following optimal set points. The passage duration and the MTX concentration should therefore be decreased to 2 days and 7.5 nM, while the initial passage viable cell density should be slightly increased to 0.225 × 10^6^ cells/mL.

## 4 CONCLUDING REMARKS

The quality by design principle determined by the FDA was applied to the early process steps of a CHO cell cultivation. Inoculum expansion process parameters were compiled and potential risks were assessed by failure mode and effect analysis. The impact of selected parameters and their interactions were investigated using a design of experiments approach in scaled down shake flasks. Multivariate data analysis was used to analyse the experimental data and calculate regression models fitting to the measured responses. Analysis of the effect‐ and response contour plots identified the passage duration as a key process parameter and its interaction effects with the initial passage viable cell density as a crucial part of the studied process. It also showed that the parameters in the inoculum expansion have a critical impact on later production process performance.

A reduced MTX concentration and passage duration improved the overall viability and growth rate of the inoculum expansion and the production process. Additionally, an opposite effect of the studied process parameters on the specific productivity was observed, which still could not compensate the discussed negative coefficients in terms of total mAb production. Further analysis of the responses resulted in the definition of a design space for the studied parameters. It showed the acceptable working range for the process, which could be expanded with decreased MTX concentration. Furthermore, it was shown to be robust for a mean initial passage viable cell density, while the optimum for the passage duration was found at the experimentally possible minimum. Based on these results, the early process steps were assessed as a critical part of the process and should consequently be investigated and analysed for newly developed processes and subsequently monitored and controlled using appropriate PAT possibilities.

Leading forward, the findings of this study will be connected to parameter effects of the production process to further increase the understanding of the process. In the course of those future studies, experiments will be carried out using the Ambr® microbioreactor platforms by Sartorius Stedim. In order to achieve further insights, product quality attributes, namely the bioactivity, glycosylation and charge variants of the produced mAb will be taken into account. Thereby, this study is considered to be the first step towards a full process characterization regarding QbD principles, highlighting the importance of early process understanding and investigation.

## CONFLICT OF INTEREST

We confirm that all corresponding authors agree with the submission and publication of this paper and that there is no conflict of interest concerning financial and personal relationships. The manuscript does not contain neither experiments using animals nor human studies. Furthermore, we confirm that the article has not been published previously by any of the authors and is not under consideration for publication elsewhere at the time of submission.

## Supporting information

Supplementary information

Supplementary information

## Data Availability

The data that supports the findings of this study are available in the supplementary material of this article.
