# Supplementary figures and images for: Implementation of QbD strategies in the inoculum expansion of a mAb production process
Source: Eng Life Sci. 2020 Dec 3;21(3-4):196–207. doi: 10.1002/elsc.202000056 (PMC7923587; doi:10.1002/elsc.202000056)

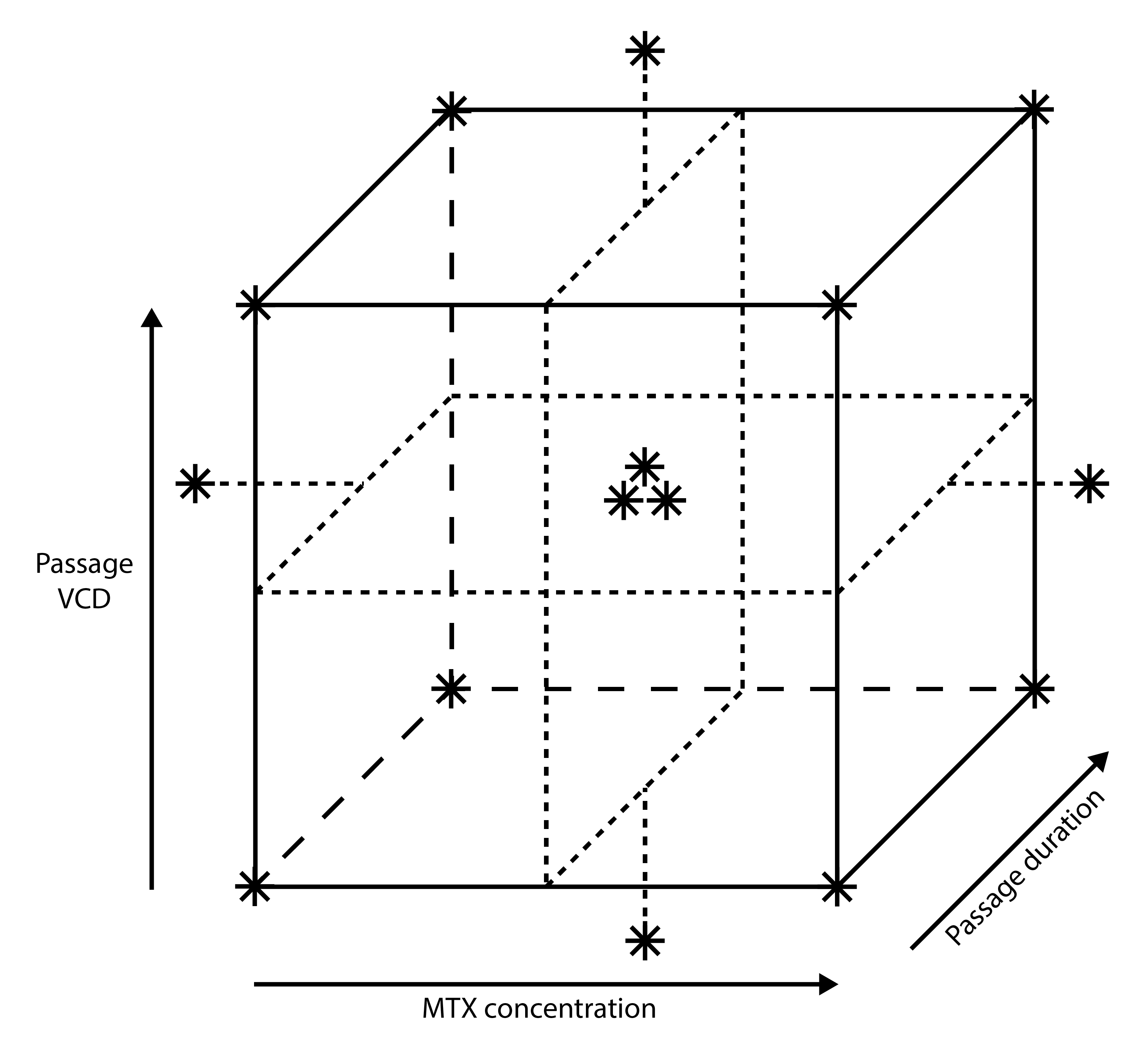

Supplement: Supplementary file 1 — Supplementary information [file ELSC-21-196-s002.png]
