# Supplementary material for: Implementation of QbD strategies in the inoculum expansion of a mAb production process
Source: Eng Life Sci. 2020 Dec 3;21(3-4):196–207. doi: 10.1002/elsc.202000056 (PMC7923587; doi:10.1002/elsc.202000056)
Supplement: Supplementary file 2 — Supplementary information [file ELSC-21-196-s001.pdf]

Supplements

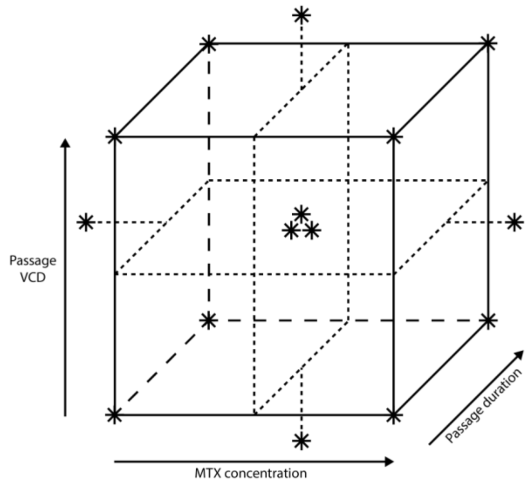

Figure S1. Schematic representation of the conducted central composite design for the Passage viable cell density, the MTX concentration and the passage duration. The eight corner points represent the -1/1 levels. The three stars in the middle of the cube represent the replicate centre-point runs (0 level). The outer stars represent the -2/2 levels for two of the three parameters.

Table S1. Risk assessment and FMEA results.

Process Parameter	Probability	Severity	Detection	RPN
**MTX concentration**	1	5	4	20
**Passage duration**	3	3	2	18
**Passage VCD**	2	2	4	16
**pH**	2	3	2	12
**Thaw temperature**	2	3	2	12
**Thaw duration**	2	2	3	12
**Osmolality**	1	3	3	9
**Media storage temperature**	1	4	2	8
**Media storage time**	1	4	2	8
**Initial VCD**	1	2	4	8
**Type of cryo conservation**	1	3	2	6
**Media temperature**	1	3	2	6
**Cell line**	1	5	1	5
**Temperature**	1	5	1	5
**Incubator CO_2**	1	4	1	4
**Agitation speed**	1	4	1	4
**Incubator humidity**	1	3	1	3
**Non-/baffled flasks**	1	3	1	3
**Working volume**	1	2	1	2

Table S2. Numerical coding of varied process parameters.

	-2	-1	0	1	2
**MTX concentration [nM]**	0	7.5	15	22.5	30
**Passage duration [d]**	-	2	3,2	5	-
**Passage VCD [x10^6 cells/ml]**	0.1	0.15	0.2	0.25	0.3

Table S3. Experimental set up and responses for the DoE.

Experiment number	MTX Concentration	Passage Duration	Passage VCD	Inoculum Viability [%]	Inoculum Growth Rate [1/d]	Production Viability [%]	Production Growth Rate [1/d]	Total mAb Titer [g/l]	Specific mAb Titer [g/l*(10^6 cells*d/ml)$^{-1}$]
**1**	-1	-1	-1	98.5	0.96	98.6	0.57	1.10	14.78
**2**	1	-1	-1	98.6	0.92	98.1	0.56	1.12	16.41
**3**	-1	1	-1	97.9	0.75	97.1	0.55	1.06	16.57
**4**	1	1	-1	98.0	0.79	97.3	0.51	1.04	17.32
**5**	-1	-1	1	98.7	0.86	98.7	0.56	1.11	17.26
**6**	1	-1	1	98.7	0.87	98.0	0.55	1.09	16.47
**7**	-1	1	1	97.4	0.69	97.3	0.49	0.98	20.87
**8**	1	1	1	96.6	0.68	96.6	0.44	0.82	23.30
**9**	-2	0	0	98.8	0.90	98.7	0.57	1.19	16.72
**10**	2	0	0	98.4	0.82	98.2	0.52	1.01	19.03
**11**	0	0	-2	98.2	0.79	97.4	0.48	0.99	20.21
**12**	0	0	2	97.9	0.72	97.4	0.48	0.90	19.58
**13**	0	0	0	98.9	0.89	98.3	0.55	1.16	17.12
**14**	0	0	0	98.5	0.88	98.2	0.57	1.06	16.11
**15**	0	0	0	98.8	0.87	98.6	0.55	1.08	17.08

Table S4. Model statistics for the inoculum expansion and production process responses

	R^2	R^2_{adj}	Q^2	p-value	Reproducibility
**Inoculum viability**	0.95	0.91	0.79	0.0001	0.90
**Inoculum growth rate**	0.99	0.98	0.96	< 0.0001	0.99
**Production viability**	0.89	0.81	0.62	0.0019	0.87
**Production growth rate**	0.99	0.90	0.69	< 0.0001	0.92
**Total mAb titer**	0.93	0.88	0.70	0.0003	0.71
**Specific mAb titer**	0.94	0.914	0.827	< 0.0001	0.90
